# Joint distraction for knee osteoarthritis: protocol for a systematic review and meta-analysis

**DOI:** 10.1186/s13643-018-0837-z

**Published:** 2018-10-15

**Authors:** En Lin Goh, Winston Choong Ngan Lou, Swathikan Chidambaram, Shaocheng Ma

**Affiliations:** 1 Faculty of Medicine, Imperial College London, South Kensington, London, SW7 2AZ UK; 2 City and Guilds Building, Biomechanics Research Group, Imperial College London, 774, 7th Floor, London, SW7 2AZ UK

**Keywords:** Osteoarthritis, Joint distraction, Knee, Systematic review, Protocol

## Abstract

**Background:**

Osteoarthritis is a degenerative joint disease that is highly prevalent worldwide. Knee osteoarthritis is the most common form of osteoarthritis and is a major cause of pain and disability. However, there remains a lack of treatments available that have demonstrated effectiveness in stopping or reversing the degenerative process. Joint distraction has emerged as a viable alternative in the treatment of knee osteoarthritis to delay the need for knee arthroplasty.

**Methods:**

An electronic search will be conducted on MEDLINE, EMBASE, Web of Science, CINAHL, Cochrane and EBSCO databases. Clinical studies investigating joint distraction for knee osteoarthritis, which reported clinical or structural outcomes including ∆WOMAC index, ∆VAS pain score and ∆joint space width will be included. Risk of bias will be assessed using the Newcastle-Ottawa Scale for observational studies and Cochrane Collaboration tool for randomised controlled trials. Quality of studies will be assessed using the modified Coleman methodology score.

**Discussion:**

This systematic review will summarise the short- and long-term clinical and structural outcomes following joint distraction for knee osteoarthritis. The findings from this review will establish the quality of currently available evidence, which will determine the need for further studies to establish the true effect size of this procedure.

**Systematic review registration:**

PROSPERO CRD42018087032

## Background

Osteoarthritis is a degenerative joint disease characterised by articular cartilage destruction, synovial membrane inflammation and subchondral bone remodelling [1]. This is the most prevalent chronic joint condition worldwide, that affects 10% of individuals over the age of 60 years [2]. Knee osteoarthritis comprises of the largest proportion of all cases and is a major cause of pain and disability, which have a significant impact on quality of life [3]. Treatment aims to relieve pain, reduce stiffness and maintain function, through the use of a combination of conservative measures including physical therapy, analgesia and surgical interventions such as arthroplasty [2]. However, currently available treatment options have failed to demonstrate effectiveness in stopping or reversing the degenerative process [4–6].

Knee arthroplasty has been shown to be effective in treating osteoarthritis in patients who have impaired quality of life despite optimal conservative treatment [7–9]. This has led to a corresponding rise in the number of procedures performed annually; it is estimated that over 40% of all procedures and 44% of revisions are performed in individuals younger than 65 years [10]. However, this procedure is associated with an increased risk of failure in these patients due to the higher functional demands [11]. In recent years, joint distraction has emerged as a viable alternative in the treatment of knee osteoarthritis [12–14]. In this surgical procedure, the two bony ends of the joint are gradually pulled over a variable distance for a period using an external fixator, thereby temporarily unloading the joint cartilage by eliminating contact between the joint surfaces [15].

Joint distraction was initially used to treat joint malalignment and contractures by preventing damage of the cartilage during forced repositioning [13]. van Valburg and colleagues were the first to observe the unexpected clinical improvements in patients with osteoarthritis following this procedure in 1995 [16]. This led to a proof-of-concept study in patients with severe ankle osteoarthritis, which demonstrated significant clinical benefits persisting for 10 years in two-thirds of the patients [16, 17]. Over the past decade, further studies have investigated the effect of knee joint distraction (KJD) in stopping and potentially reversing joint degeneration to delay knee arthroplasty in young patients, with promising results [13, 14, 18–22]. However, questions remain regarding the safety, feasibility and effectiveness of this procedure. The use of an external fixator is associated with pin tract infections, which carry the potential risk of osteomyelitis [23]. Distraction of the knee joint over a period of six to eight weeks can lead to periods of immobility, thereby increasing the risk of venous thromboembolism [20]. Additionally, KJD may interfere with activities of daily living as a result of the immobilisation, making this procedure unsuitable for certain patients. Notably, Hunziker suggested that joint distraction would be unlikely to benefit patients with osteoarthritis, as immobilisation is known to promote cartilage degeneration [24]. Consequently, the evidence-base is limited by a small number of publications on the topic. This systematic review therefore aims to assess the short- and long-term outcomes following KJD and compare this with currently available treatment modalities.

## Methods

### Protocol development and registration

This systematic review is registered in the International Prospective Register of Systematic Reviews (PROSPERO) (CRD42018087032). Literature search methods, inclusion and exclusion criteria, outcome measures and statistical analysis will be defined according to the Preferred Reporting Items for Systematic Reviews and Meta-Analyses Protocols (PRISMA-P) guidelines. Patients will not be involved in the conception, design, analysis, drafting, interpretation or revision of this research. Thus, ethics approval will not be required.

### Electronic search

The following databases will be searched: (a) MEDLINE (1946 till September week 2 2018) via OvidSP, last search on 10 September 2018; (b) MEDLINE in-process and other non-indexed citations (latest issue) via OvidSP, last search on 10 September 2018; (c) Ovid EMBASE (1974 to latest issue), last search on 10 September 2018; (d) Web of Science (latest issue), last search on 10 September 2018; (e) CINAHL Complete (latest issue), last search on 10 September 2018; Cochrane Central Register of Controlled Trials (September 2018), last search on 10 September 2018; (f) EBSCO (latest issue), last search on 10 September 2018. Search terms will use three strings linked by an AND modifier. The first string will include osteoarthritis OR arthritis OR osteoarthrosis OR arthrosis OR cartilage OR osteochondral OR degenerative joint disease; the second string: joint distraction; and the third string: knee OR tibiofemoral OR tibiofibular. Truncated search terms utilising the wildcard character and the “related articles” function will be used to broaden the search. Additionally, the references of included articles will be hand-searched to identify any additional studies.

### Study selection

All clinical studies in which invasive joint distraction was investigated as an intervention for knee osteoarthritis will be selected. The comparator arm will include patients who have undergone total knee arthroplasty, high tibial osteotomy or no intervention. In addition, all of the studies included in the systematic review will meet the following criteria: (a) radiographic signs of joint damage and primarily tibiofemoral osteoarthritis; (b) no history of inflammatory or septic arthritis; (c) valgus/varus malalignment of less than 10°; (d) reporting of at least one of the Western Ontario and McMaster Universities Osteoarthritis Index (WOMAC), visual analogue scale (VAS) or joint space width (JSW) assessed as outcome measures of the effect of the treatment; (e) article published or accepted for publication as full-length articles. No restrictions will be made on language. Non-human studies, basic science research reports, review articles, editorials, case reports, letters, conference abstracts and unpublished studies will be excluded.

### Outcome measures

Outcomes that will be assessed include change in the WOMAC index (∆WOMAC index), VAS pain score (∆VAS pain score) and JSW (∆JSW), before and after knee joint distraction procedures. Additional outcomes reported including subscales for pain, stiffness and physical function of the WOMAC index, ∆JSW as measured by radiography and magnetic resonance imaging (MRI), Knee injury and Osteoarthritis Outcome Score (KOOS) and Short Form-36 (SF-36) will be included. Short-term outcomes will be evaluated at various time points up till 1 year. Long-term outcomes of more than 1 year will be assessed at 2, 3 and 5 years.

### Risk of bias and quality assessment

The risk of bias assessment will be performed at both a study and outcome level if the latter is possible. The risk of bias of the included studies will be appraised using the Newcastle-Ottawa scale (NOS) for observational studies and the Cochrane Collaboration tool for randomised controlled trials (RCTs) [25, 26]. The quality of the studies will be assessed using the modified Coleman methodology score, a quality scoring system validated in orthopaedic and sport traumatology settings [27]. At the outcome level, the risk of bias assessment will be conducted using a funnel plot to measure publication bias. The information obtained will be used in the data synthesis to assess the quality of the reported data.

### Data extraction

The initial search will be performed by the author (W.C.N.L.) for screening. Two reviewers (E.L.G. and S.C.) will screen all the titles, abstracts and full texts for inclusion, both of whom will be blinded to authors, journals, institutional affiliations and dates of publication. Both reviewers will evaluate each selected reference independently and summarise relevant study characteristics. In the event of disagreement, a consensual decision between the two reviewers under involvement of a third independent reviewer (S.M.) will be reached. The following data items will be extracted: the year of publication, study design, sample size, country of study, type of patients, patient characteristics, joint distraction technique employed, outcome measures and conclusions. The corresponding authors of the original publications will be contacted via email in the event of insufficient data. Data will be entered into Review Manager 5.3 (Cochrane Collaboration, Oxford, UK). References will be managed using the reference management software, EndNote X7 (Clarivate Analytics).

### Data synthesis and analysis

Statistical analysis will be performed using IBM SPSS Statistics (Armonk, NY, USA). Descriptive statistics will be calculated for variables of interest. Continuous measures will be summarised with the use of means and standard deviations, while categorical data will be summarised with the use of counts and percentages. Additional subgroup analyses of patients with moderate or severe osteoarthritis, as defined by clinical and structural findings and patients undergoing different joint distraction techniques will be performed. A quantitative analysis including a meta-analysis and sensitivity analysis of subgroups will be performed if the data is sufficiently homogenous. In addition to an overall analysis, further analyses will be performed according to study design if a sufficient number of RCTs and observational studies are identified. Standard mean difference with 95% confidence intervals will be used in the analysis. Both the fixed-effects and random-effects model will be considered in the analysis of the data and the most appropriate model used to pool the results. The standard heterogeneity test, the *I*^2^ statistic, will be used to assess the consistency of the effect sizes, which indicates the percentage of the variability in effect estimates because of true between-study variance rather than within-study variance. In all cases, statistical heterogeneity will be assessed using the *I*^2^ statistic and will be categorised as low, moderate and high for an *I*^2^ statistic of above 25%, 50% and 75%, respectively. Results above 60% are considered as substantial heterogeneity. If a quantitative analysis is not appropriate, a qualitative analysis will be performed.

## Discussion

With the rise in life-expectancy and prevalence of obesity, the incidence of osteoarthritis is projected to increase further over the next decade [28]. Given the limitations of current treatment modalities, the outcomes of KJD warrant a detailed examination in the literature. Several hypotheses explaining the mechanism by which joint distraction reverses the degenerative process seen in osteoarthritis have been proposed, notably through the inhibition of pro-inflammatory cytokines, metalloproteinases and activation of chondrocytes [29, 30]. The present systematic review will enable us to draw conclusions regarding the short- and long-term clinical and structural outcomes following joint distraction for knee osteoarthritis. Conducting a systematic review of the literature will provide a summary of the existing findings on this topic and critical appraisal of the risk of bias and methodological quality of the currently available evidence. These will be essential in designing of future studies to establish the true effect size of this procedure.

## Abbreviations

JSW: Joint space width
KJD: Knee joint distraction
PRISMA: Preferred items for reporting systematic review and meta-analysis
PROSPERO: International Prospective Register of Systematic Reviews
VAS: Visual analogue scale
WOMAC: Western Ontario and McMaster Universities Osteoarthritis Index
